# Analysis of Soil Microbial Features in a Rice Paddy Field with High Methane Emissions

**DOI:** 10.1264/jsme2.ME25044

**Published:** 2025-11-27

**Authors:** Yoriko Sakai, Ichiro Uezono, Makoto Shibuya, Noriko Oura, Shigeto Sudo

**Affiliations:** 1 Institute for Agro-Environmental Sciences, National Agriculture and Food Research Organization (NARO), Tsukuba, Ibaraki, Japan; 2 Kagoshima Prefectural Institute of Agricultural Development, Minamisatsuma, Kagoshima, Japan; 3 Akita Agricultural Experiment Station, Akita, Akita, Japan

**Keywords:** rice paddy field, *mcrA*, “*Candidatus* Methanoperedens”, methanotroph

## Abstract

The soil microbial communities in two Japanese paddy fields were compared: site PA, which emits methane at the domestic average level, and site PX, which emits more than five times that amount. The transcription levels of methyl-coenzyme M reductase (*mcrA*) significantly increased during peak methane emissions at site PX, but not at site PA. The anaerobic methanotroph “*Candidatus* Methanoperedens” exhibited low activity and abundance exclusively at site PX. The genus *Methanoregula* was the most active methanogen at both sites; however, the dominant methanotrophs differed, with *Methylocystis* dominating at site PA and *Methylomonas* at site PX.

Methane is the second most abundant greenhouse gas emitted from natural sources and anthropogenic activities. In paddy fields, organic matter in soil is decomposed and metabolized into low-molecular-weight materials through microbial metabolism, and some may be used as substrates for methanogens (Alpana *et al.*, 2017). Methane produced by methanogens is partially consumed by methane-oxidizing microorganisms, while the remainder is released into the atmosphere (Le Mer and Roger, 2001).

The amount of reducible ions in the soil significantly impacts methane emissions in paddy fields. When paddy field soil becomes flooded, substances are reduced in the order of thermodynamic favorability (Lovley, 1991). The genera *Geobacter* and *Anaeromyxobacter* are the main iron-reducing bacteria in paddy field soils (Hori *et al.*, 2010; Masuda *et al.*, 2024). In incubation experiments using a paddy field soil, *Geobacter* spp. used iron as an electron acceptor to oxidize acetic acid, thereby decreasing soil methane production (Hori *et al.*, 2010).

A project investigating greenhouse gas emissions from agricultural fields in Japan (https://www.maff.go.jp/j/seisan/kankyo/tuti_chyosa.html) (Oura *et al.*, unpublished) revealed that some paddy fields emitted methane at markedly higher levels annually than the national average. In the present study, we selected two paddy sites from this project to analyze soil microbial communities and identify candidate microbial groups linked to elevated methane emissions from rice fields. The composition and activity of soil microbes serve as an indicator of the environmental conditions present at each site.

Table S1 shows details of the two experimental sites (PA and PX). Site PA emits methane at the domestic average level, whereas site PX emits more than five times this amount. Rice straw-removed plots (N, no rice straw, removed after harvest) and rice straw-plowed plots (RS, 0.5‍ ‍kg m^–2^) were run in triplicate at both sites. Soil samples were obtained at two time points: drained (D) and waterlogged (W). The soil sampling procedure is described in the supplemental material, Tx S1. Nucleic acids were extracted from soil samples according to a previously reported technique with some modifications, particularly to reduce DNA fragmentation. The copy numbers of the methyl-coenzyme M reductase (*mcrA*) gene and archaeal and bacterial 16S rRNA genes, as well as their transcripts, were quantified using a real-time PCR system (StepOne; Thermo Fisher Scientific). Nucleotide sequences were analyzed using a next-generation sequencer. The details of each procedure are shown in Tx S1.

The results of the quantitative PCR anal­ysis showed that the copy numbers of the *mcrA* and 16S rRNA genes were lower during peak methane emissions (W) than before flooding (D) at site PA, but remained nearly unchanged at site PX (Fig. 1). Conversely, the transcription levels of the *mcrA* and archaeal 16S rRNA genes were significantly higher during peak methane emissions than before flooding at site PX, but remained unchanged or decreased at site PA. These results suggest that the soil at site PX maintained microbial populations under reducing conditions after flooding, which prevented a decrease in microbial abundance while significantly activating archaeal groups, including methanogens. The copy numbers and transcription levels of these genes typically fluctuate throughout the rice growing season (*e.g.*, Watanabe *et al.*, 2009; Itoh *et al.*, 2013; Lee *et al.*, 2014), and, thus, further studies are required to confirm the present results. Similar findings supporting this view were obtained in a subsequent survey conducted at site PX (Sakai *et al.*, unpublished).

An amplicon anal­ysis of the soil at site PA revealed that most of the *mcrA* genes expressed in pre-flooded PA soil (D) were from “*Candidatus* Methanoperedens” (formally ANME-2d) (Fig. 2). This genus is closely related to the genus *Methanosarcina*, but is known to oxidize methane in an anaerobic environment using the *mcrA* gene in the reverse reaction of methane production (Evans *et al.*, 2019). The abundance of “*Ca.* Methanoperedens” was negligible at site PX. Quantitative PCR yielded similar results (Fig. S1). “*Ca.* Methanoperedens” in paddy fields was previously exami­ned in Italy (Vaksmaa *et al.*, 2017) and China (Shen *et‍ ‍al.*, 2021) and was also detected in the field soil samples‍ ‍of several countries, including Japan (*e.g.*, Fernández-Baca *et al.*, 2021; Sakoda *et al.*, 2022). Therefore, “*Ca.* Methanoperedens” appears to be ubiquitous in paddy soil, and its absence may be one of the factors increasing methane emissions at site PX. Among methanogens, the group with the highest *mcrA* gene transcription level was common to both sites and belonged to the genus *Methanoregula* (Fig. 2).

The amplicon anal­ysis of 16S rRNA genes showed that the abundance and activity of the phyla *Pseudomonadota* (including most parts of the former genus *Proteobacteria*), *Acidobacteriota*, and *Chloroflexota* were high at both sites (Fig. S2A), which is consistent with previous findings (*e.g.*, Lee *et al.*, 2014; Vaksmaa *et al.*, 2017). Aerobic methanotrophs were detected at high ratios at both sites, although the dominant group differed (Fig. 3A and S3). The genus *Methylomonas*, which was mainly detected at site PX, is a methanotroph that prefers higher concentrations of methane and higher pH (Ho *et al.*, 2013; Kambara *et al.*, 2022). This finding is consistent with the differences observed between the two sites. Archaeal 16S rRNA genes accounted for a small percentage of total 16S rRNA genes (Fig. S2B), with *Thermoproteota* (mostly comprising *Nitrososphaeria* and *Bathyarchaeia*) and *Nanoarchaeota* accounting for the majority, while *Methanobacteriota*, *Thermoplasmatota*, and *Halobacteriota*, to which the main methanogens belonged, accounted for less than 10% of archaeal 16S rRNA genes. The copy number and transcripts of 16S rRNA genes from *Nanoarchaeota* significantly increased during peak methane emissions (W) at site PX.

Contrary to expectations, genes from the family *Geobacteraceae*, including *Geobacter* and its relatives, which are known as iron-reducing bacteria, were transcribed at higher levels under flooding (W) at site PX than at site PA (Fig. 3A), despite the lower content of extractable ions at site PX (Table S1). When the breakdown of *Geobacteraceae* sequences detected in the present study was exami­ned, more than half were “unclassified” (Fig. 3B). The operational taxonomic units (OTU, 98%) of these “unclassified” sequences showed that the representative sequence of OTU2 was 98% identical to the sequence of *Geobacter metallireducens* GS-15, a representative strain of direct interspecies electron transfer (DIET) (Rotaru *et al.*, 2014) (Fig. S4B). The sequence of OTU3 was 99% identical to the sequence of *Oryzomonas japonica* Red96, which exhibits the ability to fix N_2_ (Masuda *et al.*, 2024). Therefore, some of the electron flow at site PX may be used by DIET and N_2_ fixation. DIET between the genera *Geobacter* and *Methanothrix* has been indicated in rice paddies in the United States (Holmes *et al.*, 2017).

The plowing of rice straw into sites PA and PX enhanced methane emissions (Oura *et al.*, unpublished), as previously reported (*e.g.*, Yagi and Minami, 1990); however, the shift in the soil microbial organization was smaller than that observed among locations differing in soil type and climate (Fig. S5). Locations and soil types have been identified as important factors affecting the soil bacterial structure and activity (*e.g.*, Bao *et al.*, 2012).

Excessive methane production at site PX may have been due to a lack of microbially available iron in the soil. However, no other rice paddy fields emitted a similar amount of methane after the completion of iron reduction, making it difficult to attribute this solely to iron. While we detected several important microbial groups in the present study, their specific roles in methane metabolism warrant further investigation.

Sequence data were deposited in the DNA Data Bank under BioProject ID PRJDB15976.

## Conflicts of Interest

The authors declare that there are no conflicts of interest.

## Citation

Sakai, Y., Uezono, I., Shibuya, M., Oura, N., and Sudo, S. (2025) Analysis of Soil Microbial Features in a Rice Paddy Field with High Methane Emissions. *Microbes Environ ***40**: ME25044.

https://doi.org/10.1264/jsme2.ME25044

## Supplementary Material

Supplementary Material

## Figures and Tables

**Fig. 1.**
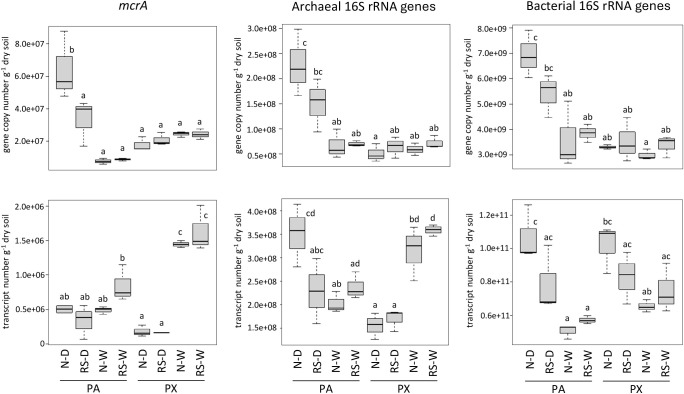
Comparison of copy numbers for genes and transcripts of *mcrA*, archaeal 16S rRNA, and bacterial 16S rRNA in PA and PX soil samples. N, rice straw removed; RS, rice straw plowed; D, drained sample; W, water-logged sample. Lowercase letters a, b, c, and d indicate significant differences in copy numbers within each gene or transcript (*P*<0.05).

**Fig. 2.**
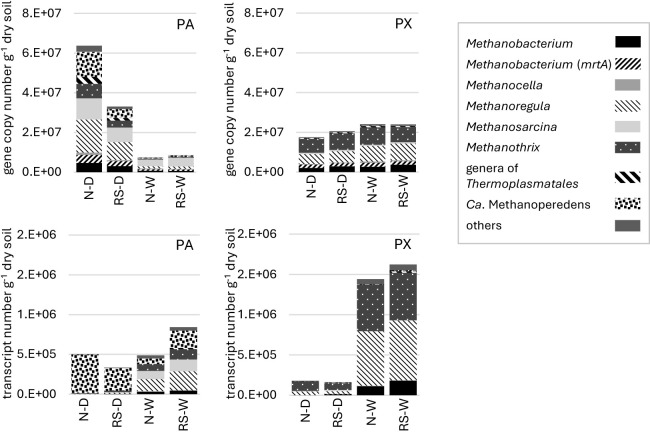
Abundance and transcript counts of *mcrA* genes from each genus in soil samples from sites PA and PX, calculated using amplicon sequencing and real-time PCR data. N, rice straw removed; RS, rice straw plowed; D, drained sample; W, water-logged sample.

**Fig. 3.**
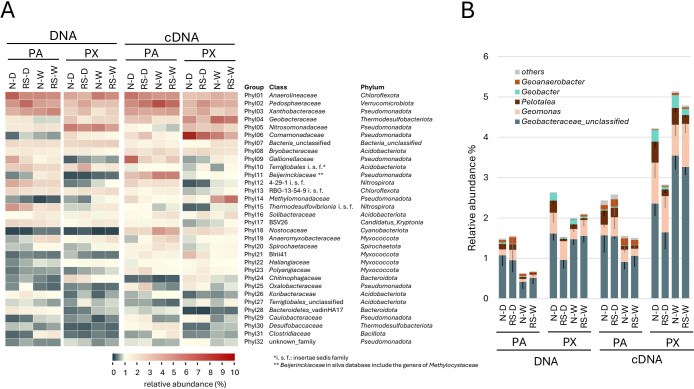
Heatmap showing the relative abundance of sequences from total 16S rRNA genes and transcripts in each soil sample from sites PA and PX (A), and a bar graph showing the genus level ratio of *Geobacteraceae* within total 16S rRNA genes sequences. (B). The heat map shows the 30 most abundant families. N, rice straw removed; RS, rice straw plowed; D, drained sample; W, water-logged sample.

## References

[B1] Alpana, S., Vishwakarma, P., Adhya, T.K., Inubushi, K., and Dubey, S.K. (2017) Molecular ecological perspective of methanogenic archaeal community in rice agroecosystem. Sci Total Environ 596–597: 136–146. 10.1016/j.scitotenv.2017.04.011 28431358

[B2] Bao, Z., Ikunaga, Y., Matsushita, Y., Morimoto, S., Takada-Hoshino, Y., Okada, H., et al. (2012) Combined anal­yses of bacterial, fungal and nematode communities in Andosolic agricultural soils in Japan. Microbes Environ 27: 72–79. 22223474 10.1264/jsme2.ME11281 PMC4036027

[B4] Evans, P.N., Boyd, J.A., Leu, A.O., Woodcroft, B.J., Parks, D.H., Hugenholtz, P., et al. (2019) An evolving view of methane metabolism in the Archaea. Nat Rev Microbiol 17: 219–232. 30664670 10.1038/s41579-018-0136-7

[B5] Fernández-Baca, C.P., Rivers, A.R., Kim, W., Iwata, R., McClung, A.M., Roberts, D.P., et al. (2021) Changes in rhizosphere soil microbial communities across plant developmental stages of high and low methane emitting rice genotypes. Soil Biol Biochem 156: 108233.

[B6] Ho, A., Kerckhof, F.M., Luke, C., Reim, A., Krause, S., Boon, N., et al. (2013) Conceptualizing functional traits and ecological characteristics of methane-oxidizing bacteria as life strategies. Environ Microbiol Rep 5: 335–345. 23754714 10.1111/j.1758-2229.2012.00370.x

[B7] Holmes, D.E., Shrestha, P.M., Walker, D.J.F., Dang, Y., Nevin, K.P., Woodard, T.L., et al. (2017) Metatranscriptomic evidence for direct interspecies electron transfer between *Geobacter* and *Methanothrix* species in methanogenic rice paddy soils. Appl Environ Microbiol 83: e00223-17. 28258137 10.1128/AEM.00223-17 PMC5394310

[B8] Hori, T., Müller, A., Igarashi, Y., Conrad, R., and Friedrich, M.W. (2010) Identification of iron-reducing microorganisms in anoxic rice paddy soil by 13C-acetate probing. ISME J 4: 267–278. 19776769 10.1038/ismej.2009.100

[B9] Itoh, H., Ishii, S., Shiratori, Y., Oshima, K., Otsuka, S., Hattori, M., and Senoo, K. (2013) Seasonal transition of active bacterial and archaeal communities in relation to water management in paddy soils. Microbes Environ 28: 370–380. 24005888 10.1264/jsme2.ME13030 PMC4070958

[B10] Kambara, H., Shinno, T., Matsuura, N., Matsushita, S., Aoi, Y., Kindaichi, T., et al. (2022) Environmental factors affecting the community of methane-oxidizing bacteria. Microbes Environ 37: ME21074. 35342121 10.1264/jsme2.ME21074 PMC8958294

[B11] Le Mer, J., and Roger, P. (2001) Production, oxidation, emission and consumption of methane by soils: a review. Eur J Soil Biol 37: 25–50.

[B12] Lee, H.J., Kim, S.Y., Kim, P.J., Madsen, E.L., and Jeon, C.O. (2014) Methane emission and dynamics of methanotrophic and methanogenic communities in a flooded rice field ecosystem. FEMS Microbiol Ecol 88: 195–212. 24410836 10.1111/1574-6941.12282

[B13] Lovley, D.R. (1991) Dissimilatory Fe(III) and Mn(IV) reduction. Microbiol Mol Biol Rev 55: 259–287. 10.1128/mr.55.2.259-287.1991 PMC372814 1886521

[B14] Masuda, Y., Mise, K., Xu, Z., Zhang, Z., Shiratori, Y., Senoo, K., and Itoh, H. (2024) Global soil metagenomics reveals distribution and predominance of *Deltaproteobacteria* in nitrogen-fixing microbiome. Microbiome 12: 95. 38790049 10.1186/s40168-024-01812-1 PMC11127431

[B15] Rotaru, A.-E., Shrestha, P.M., Liu, F., Shrestha, M., Shrestha, D., Embree, M., et al. (2014) A new model for electron flow during anaerobic digestion: direct interspecies electron transfer to *Methanosaeta* for the reduction of carbon dioxide to methane. Energy Environ Sci 7: 408–415.

[B16] Sakoda, M., Tokida, T., Sakai, Y., Senoo, K., and Nishizawa, T. (2022) Mitigation of paddy field soil methane emissions by Betaproteobacterium *Azoarcus* inoculation of rice seeds. Microbes Environ 37: ME22052. 36517028 10.1264/jsme2.ME22052 PMC9763044

[B17] Shen, L., Yang, W., Yang, Y., Liu, X., Tian, M., Jin, J., et al. (2021) Spatial and temporal variations of the community structure and abundance of *Candidatus* Methanoperedens nitroreducens-like archaea in paddy soils. Eur J Soil Biol 106: 103345.

[B18] Vaksmaa, A., van Alen, T.A., Ettwig, K.F., Lupotto, E., Valè, G., Jetten, M.S.M., and Lüke, C. (2017) Stratification of diversity and activity of methanogenic and methanotrophic microorganisms in a nitrogen-fertilized Italian paddy soil. Front Microbiol 8: 2127. 29180985 10.3389/fmicb.2017.02127 PMC5693880

[B19] Watanabe, T., Kimura, M., and Asakawa, S. (2009) Distinct members of a stable methanogenic archaeal community transcribe *mcrA* genes under flooded and drained conditions in Japanese paddy field soil. Soil Biol Biochem 41: 276–285.

[B20] Yagi, K., and Minami, K. (1990) Effect of organic matter application on methane emission from some Japanese paddy fields. Soil Sci Plant Nutr 36: 599–610.

