# Supplementary material for: Analysis of Soil Microbial Features in a Rice Paddy Field with High Methane Emissions
Source: Microbes Environ. 2025 Nov 27;40(4):ME25044. doi: 10.1264/jsme2.ME25044 (PMC12727204; doi:10.1264/jsme2.ME25044)
Supplement: Supplementary file 1 — Supplementary Material [file 40_25044_s1.pdf]

Supplementary information

Microbes Environ. 40(4), 2025 https://www.jstage.jst.go.jp/browse/jsme2 doi:10.1264/jsme2.ME25044

Short Communication

Analysis of Soil Microbial Features in a Rice Paddy Field with High Methane Emissions

Yoriko Sakai[1]*, Ichiro Uezono[2], Makoto Shibuya[3], Noriko Oura[1], and Shigeto Sudo[1]

[1]Institute for Agro-Environmental Sciences, National Agriculture and Food Research Organization (NARO), Tsukuba, Ibaraki, Japan; [2]Kagoshima Prefectural Institute of Agricultural Development, Minamisatsuma, Kagoshima, Japan; and [3]Akita Agricultural Experiment Station, Akita, Akita, Japan

Tx S1 Supplementary information on experimental methods

1) Sampling procedures

Soil samples were obtained from the fields at depths ranging from 0 to 0.1 m without surface soil at two time points: drained samples (D) and water-logged samples (W). The D samples of site PA and PX were sampled on 8th May and 9th June, and the W samples were collected on 24th June and 16th September, respectively, in 2014. The date of waterlogged sampling was decided based on previous investigations into maximum methane emission. Scooping was collected in sample D, which was transported to the laboratory in plastic bags at room temperature. With 50 mL open-ended plastic syringes, sample W was obtained as the soil core between the rows of rice plants. To prevent oxidation by air, the syringes containing the soil samples were sealed with silicon stoppers and transported to the laboratory in an ice-water bath. Two cores from each plot were thoroughly combined in a plastic bag and used as a single sample for each plot. Soil samples (0.4 g) were weighed and deposited in 2 mL vials as quickly as possible before being kept at -80 °C until further usage.

2) Procedures for preparing nucleic acids

Nucleic acids were extracted from the soil samples using the method described by Breidenbach et al. (2015) with some modifications. Briefly, 1 mL of ice-cold RNA-later ICE (Thermo Fisher Scientific, USA) was added to a 0.4 g frozen soil sample in a 2 mL screw-cap tube and then kept at -20 °C overnight with mixing by tapping several times. This tube was then centrifuged for 1 min at 20,000 × g, and the resultant supernatant was removed. Beads from a Matrix E tube (MP Biomedicals, USA) were transferred to a 2 mL tube, and the extraction procedures were conducted three times with an extraction buffer (Breidenbach et al. 2015) under different strengths of shaking to reduce damage to the microbial nucleic acid. For the first extraction, 750 µL of the buffer was added to the soil sample in the 2 mL tube, and the tube was shaken with a bead-beating instrument (FastPrep, MP Biomedicals, USA) for 5 s at 4.0 $m.s^{-1}$. After centrifuging for 5 min. At 20,000 g, the supernatant was transferred to a new tube and immediately mixed well with 0.5 mL of phenol-chloroform-isoamyl alcohol (PCI, 25:24:1). For the second and third extractions, 550 µL of the extraction buffer was added to the soil sample in the 2 mL tube and shaken for 10 s at 4.5 $m.s^{-1}$ and 15 s at 5.5 $m.s^{-1}$, respectively. Each supernatant was mixed with PCI in a new tube. The tubes containing the supernatants and PCI were centrifuged, and the upper layers of the triplicate extractions were combined. Crude nucleic acids in the supernatant were precipitated with a 0.1 volume of 3 M sodium acetic acid and a 0.6 volume of isopropanol and then washed with 70% ethanol. Crude nucleic acid pellets in each tube were dissolved in water and purified using a MicroSpin S-400 HR Column (Cytiva, USA). Half of this solution was diluted 100 times with 1:10 TE buffer as the DNA template, and the other half was used to prepare the cDNA template. The DNA in the solution was removed using

RNase-free DNase (RQ1, Promega, USA), and the resultant RNA solution was purified using a QIAquick PCR purification kit (QIAGEN, Netherlands) (Töwe et al., 2011). A portion of the RNA solution was used to synthesize cDNA using PrimeScript reverse transcriptase (Takara, Japan). The obtained cDNA solution was purified using a PCR purification kit and diluted five times with 1:10 TE buffer as the cDNA template. For the soil samples from PA, 100 µL of casein solution (5% in the extraction buffer) (Ikeda *et al.*, 2008) and 650 µL of the extraction buffer were replaced with 750 µL of the extraction buffer at the first extraction. For the soil samples from PX, the addition of RNA-later ICE was omitted, as the reagent significantly inhibited nucleic acid extraction, and RNA extraction was improved without the reagent. These extraction and transcription procedures were conducted in duplicate for soil samples from each plot.

3) Procedures for quantitative PCR

PCR mixtures were prepared with TaKaRa Ex-Taq Hot Start Version, PCR buffer, dNTPs attached to the polymerase, primers (Table S2), 2 µL of DNA or cDNA template, 0.2 µL of SYBR green I solution (1000 times dilution with water, SYBR Green I Nucleic Acid Gel Stain, Lonza, Switzerland), 0.3 µL of Rox reference dye (Thermo Fisher Scientific), and 0.3 µL of bovine serum albumin solution (20 mg mL^{-1}, Takara), with a total volume of 15 µL. Table S2 shows the reaction program for the qPCR experiments. The cloned vectors were linearized, gradually diluted, and used as standards. Statistical analysis of copy numbers in each soil sample was performed using the Tukey–Kramer test in R (ver. 4.0.3).

4) Procedures for the next generation sequencing

Partial nucleotide sequences of *mcrA* were amplified by PCR from the combined template of DNA and cDNA from triplicate plots for each treatment. The PCR products were adapted to the barcode sequences used in the second PCR. PCR mixtures were prepared with TaKaRa Ex-Taq Hot Start Version, PCR buffer, dNTPs attached to the polymerase, primers (Table S2), and 2 µL of DNA or cDNA template, for a total volume of 20 µL. Details of the primers and PCR programs are presented in Table S2. The 1st and 2nd PCR reactions were conducted in triplicate for each template to reduce PCR drift. The PCR products were combined, purified, and used as templates for nucleotide sequence analysis with a 454 GS Junior System sequencer (Roche Applied Science, Germany) per the manufacturer's instructions. Partial nucleotide sequences of rDNA genes were also analyzed as *mcrA* genes, with some modifications. The templates for the DNA and cDNA from the triplicate plots of each treatment were analyzed separately without combining them, and the PCR reactions were conducted in single without triplicate. The rDNA sequences were analyzed with an Illumina MiSeq sequencer using the MiSeq Reagent Kit v2 (500 cycles) (Illumina, USA). Sequence data were processed using the Mothur software v. 1.48.2 (Schloss et al.*,* 2009). In rDNA sequence processing, sequence data that had fewer than four mismatches with the primer sequences and no mismatches with the barcode sequences were

passed to include broader organisms with some quality control. Then, 400 bp or fewer sequences (after removing primer and adapter sequences) were applied for the analysis in this study. The resultant rDNA sequences were classified according to the rDNA sequence dataset SILVA v138.2 (https://mothur.org/wiki/silva_reference_files/). Although universal primers were used (Table S2), the PCR conditions and cut-off values for amplicon sequence processing probably reduced the ratio of 18S rDNA sequences whose PCR products were longer than those of 16S rDNA. The ratio of the sequences classified as unknown or eukaryotic was less than 0.3% (data not shown) and was negligible. The sequences annotated as chloroplast, mitochondria, unknown, or Eukaryota were removed before analysis. Reference sequences for *mcrA* genes and 16S rDNA genes were obtained from the National Center for Biotechnology Information (https://www.ncbi.nlm.nih.gov/). Phylogenetic trees were constructed using MEGA 11 software (Tamura et al., 2021).

Table S1 Locations, soil properties and field management of the fields

	PA	PX
City, prefecture	Akita, Akita	Minamisatsuma, Kagoshima
Latitude, longitude	39° 57′ N, 140° 19′ E	31° 48′ N, 130° 34′ E
Soil texture		
coarse sand 2-0.2 mm	14.4	43.4
fine sand 0.2-0.02 mm	21.7	38.6
silt 0.02-0.002 mm	30.7	10.8
cray 0.002 mm >	33.2	7.2
Soil pH(H_2O) †	5.3	6.0
Soil C% †	4.7	0.73
Soil N% †	0.33	0.06
Extractable-Fe (g kg^{-1}) ‡	10.5	3.0
Fertilizer		
N (N kg ha^{-1})	9.0	7.0
P (P_2O_5 kg ha^{-1})	3.7	6.0
K (K_2O kg ha^{-1})	3.7	7.7
Oryza sativa L., cultivor	Akitakomachi	Hinohikari
Date or month of ivent		
rice-straw plawing	April in 2014	Novemver in 2013
water filling	14/5/2014	13/6/2014
planting	20/5/2014	18/6/2014
mid-season drainige	end of June	end of August

† Data are originated from the report of basic survey project for calculation of gas emission from agricultural land soils (Ministry of Agriculture, Forestry and Fisheries, 2014 in Japanese).

‡ Citrate-dithionite extractable iron was analyzed by the downschaled procedure of Holmgren (1967).

Table S2 Reaction programs, standards and primers for quantitative PCR (qPCR) and preparing template for amplicon sequence

target gene	experiment	origin of constructing standard plasmid for qPCR	reaction program	primer sequence (5'-3')[†]【primer name, concentration in reaction mixture μM】	reference for primer
total *mcrA*	qPCR	*Methanosarcina acetivorans* strain C2A (NBRC 100939G, NBRC, NITE, Kisarazu, Japan)	94°C for 3 min; 40 cycles of 94°C for 15 s, 49°C for 30 s, 72°C for 30 s	forward; TGTCiGGiGGiGTMGGiTTYAC【ME3MF_I, 1】<br>reverse; TCATBGCRTAGTTNGGRTAGT【ME2mod, 1】	Nunoura et al. (2008), modified in this study<br>Mori *et al*. (2012)
total *mcrA*	1st PCR for preparing template	—	94°C for 3 min; 35 cycles of 94°C for 15 s, 49°C for 30 s, 72°C for 60 s; 72°C for 3 min	same as qPCR	—<br>—
	2st PCR for preparing template	—	94°C for 3 min; 15 cycles of 94°C for 15 s, 49°C for 30 s, 72°C for 60 s; 72°C for 3 min	forward; [adapter for 454 sequence]TGTCiGGiGGiGTMGGiTTYAC【ME3MF_I_454, 0.3】<br>reverse; [adapter for 454 sequence][bar code]TCATBGCRTAGTTNGGRTAGT【ME2mod_454, 0.3】	—<br>—
mcrA of "*Candidatus* Methanoperedens"	qPCR	*mcrA* fragment from soil	94°C for 3 min; 45 cycles of 94°C for 15 s, 62°C for 15 s, 72°C for 15 s	forward; AAAGTGCGGAGCAG CAATCACC【McrA159F, 0.1】<br>reverse; TCGTCCCATTCCTGCTG CATTGC【McrA345R, 0.1】	Vaksmaa *et al*. (2017)<br>Vaksmaa *et al*. (2017)
Archaeal 16S rDNA	qPCR	*Methanosarcina barkeri* strain MS (NBRC 100474G, NBRC)	94°C for 3 min; 45 cycles of 94°C for 15 s, 61°C for 15 s, 72°C for 15 s	forward; AGGAATTGGCGGGRGRGCAC【arc915fmc, 0.1】<br>reverse; GCCATGCACCWCCTCT【arc1059r, 0.1】	Shakya *et al.* (2013)<br>Shakya *et al*. (2013)
Bacterial 16S rDNA	qPCR	*Pseudomonas putida* strain KT2440	94°C for 3 min; 45 cycles of 94°C for 15 s, 61°C for 15 s, 72°C for 15 s	forward; ACTCCTACGGGDGGCWGCAG【Eub338mc, 0.1】<br>reverse; ATTACCGCGGCTGCTGG【Eub518, 0.1】	Shakya *et al*. (2013)<br>Shakya *et al.* (2013)
Universal rDNA[‡]	1st PCR for preparing template	—	94°C for 3 min; 15 cycles of 94°C for 15 s, 45°C for 15 s, 72°C for 30 s; 72°C for 3 min	forward; GYCAGCMGCCGCGGTAA【Univ517F, 1】<br>reverse; CCGYCAATTCMTTTRAGTTT【907R, 1】	Armitage *et al.* (2012), modified in this study<br>Armitage *et al*. (2012)
	2st PCR for preparing template	—	94°C for 3 min; 15 cycles of 94°C for 15 s, 45°C for 15 s, 72°C for 30 s; 72°C for 3 min	forward; [adapter for Miseq sequence]GYCAGCMGCCGCGGTAA【Univ517F_Miseq, 0.3】<br>reverse; [adapter for Miseq sequence]CCGYCAATTCMTTTRAGTTT【907R_Miseq, 0.3】	—<br>—
	3rd PCR for preparing template	—	95°C for 3 min; 15 cycles of 95°C for 30 s, 55°C for 30 s, 72°C for 30 s; 72°C for 5 min	forward; Index primer (Nextera XT Index Kit, Illumina)<br>reverce; Index primer (Nextera XT Index Kit, Illumina)	—<br>—

† Sequences of adapters and bar codes for 454 and Miseq apmlicon sequence followed manufactures instructions.

‡ Sequences of bacterial rDNA were collected through a software Mothur and used in this study.

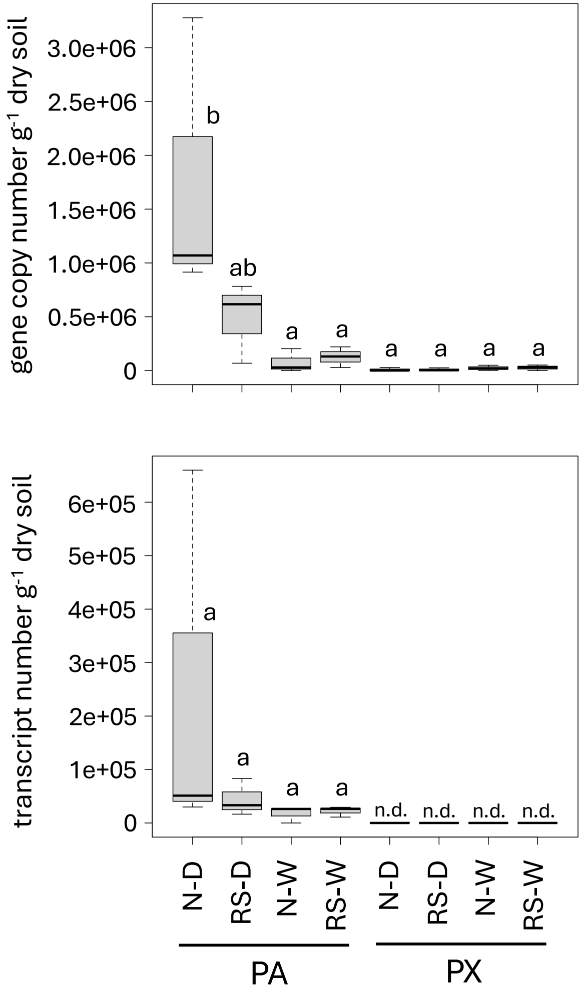

Fig. S1 Comparison of the copy numbers of *mcrA* genes and transcripts from "*Candidatus* Methanoperedens" in PA and PX soil samples. N, rice straw removed; RS, rice straw plowed; D, drained sample; W, water-logged sample. "n.d." means not detected. Lowercase letters a and b indicate significant differences in copy numbers within each gene or transcript ($P < 0.05$).

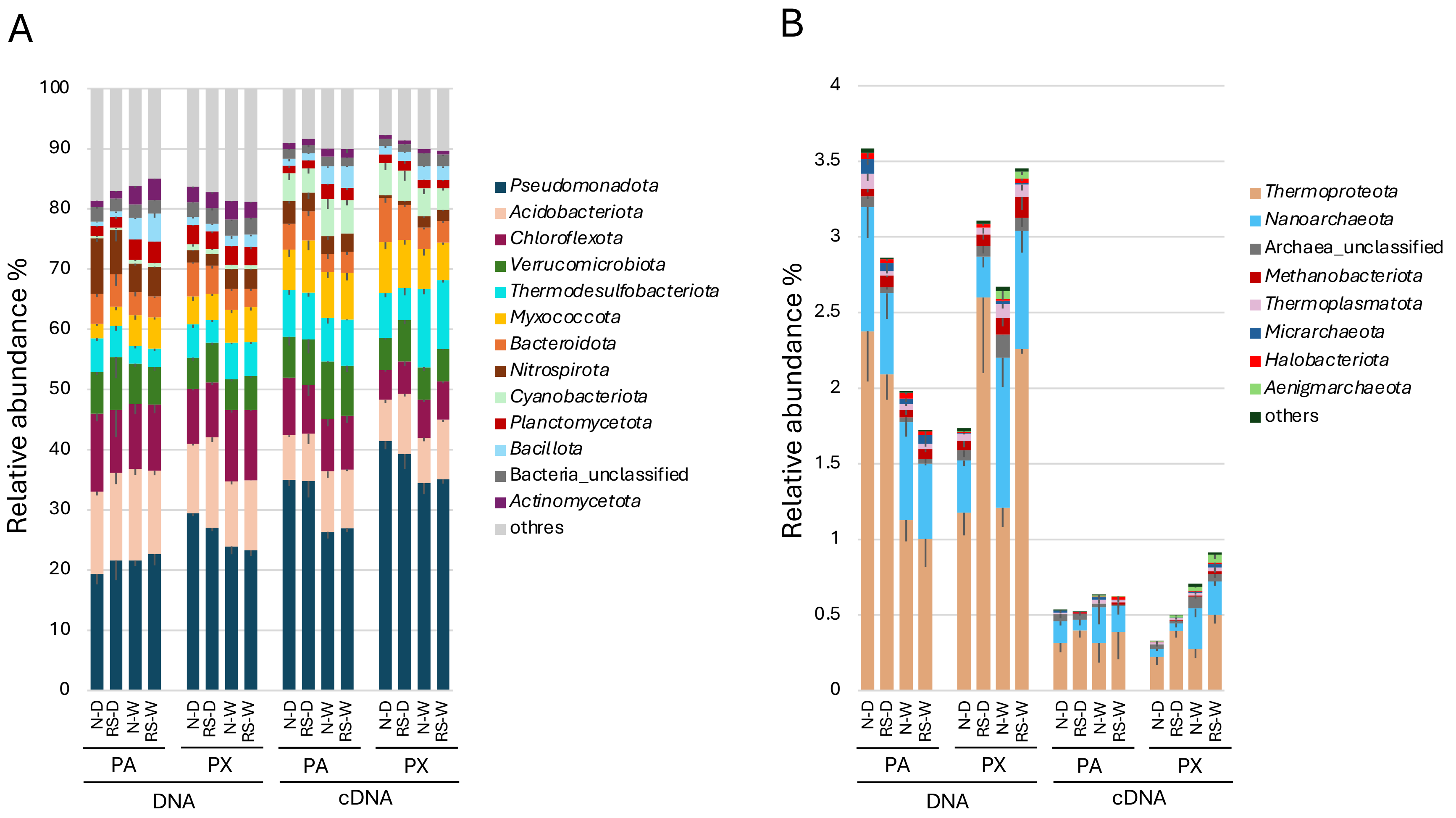

Fig. S2 Relative abundance of the top 12 most abundant bacterial phyla (A) and the top 7 most abundant archaeal phyla (B) in the amplicon analysis of the total 16S rDNA sequences. N, rice straw removed; RS, rice straw plowed; D, drained sample; W, water-logged sample.

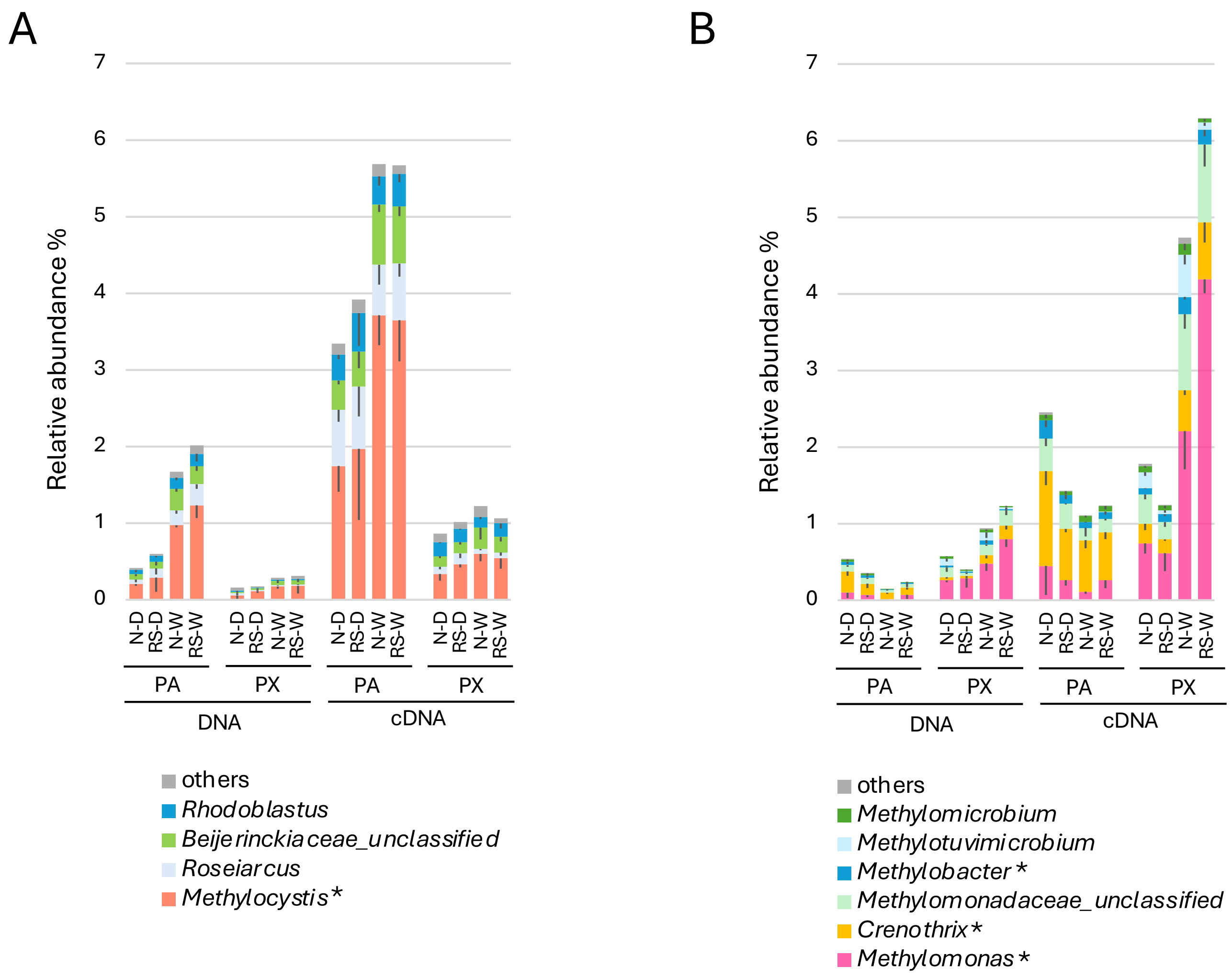

Fig. S3 Bar graphs of *Beijerinckiaceae* (A) and *Methylomonadaceae* (B) with genus-level ratio. The abundance shows the ratio against the total 16S rDNA sequences in each sample. N, rice straw removed; RS, rice straw plowed; D, drained sample; W, water-logged sample. Silva database was used for this classification. * shows the genera known as methanotrophs.

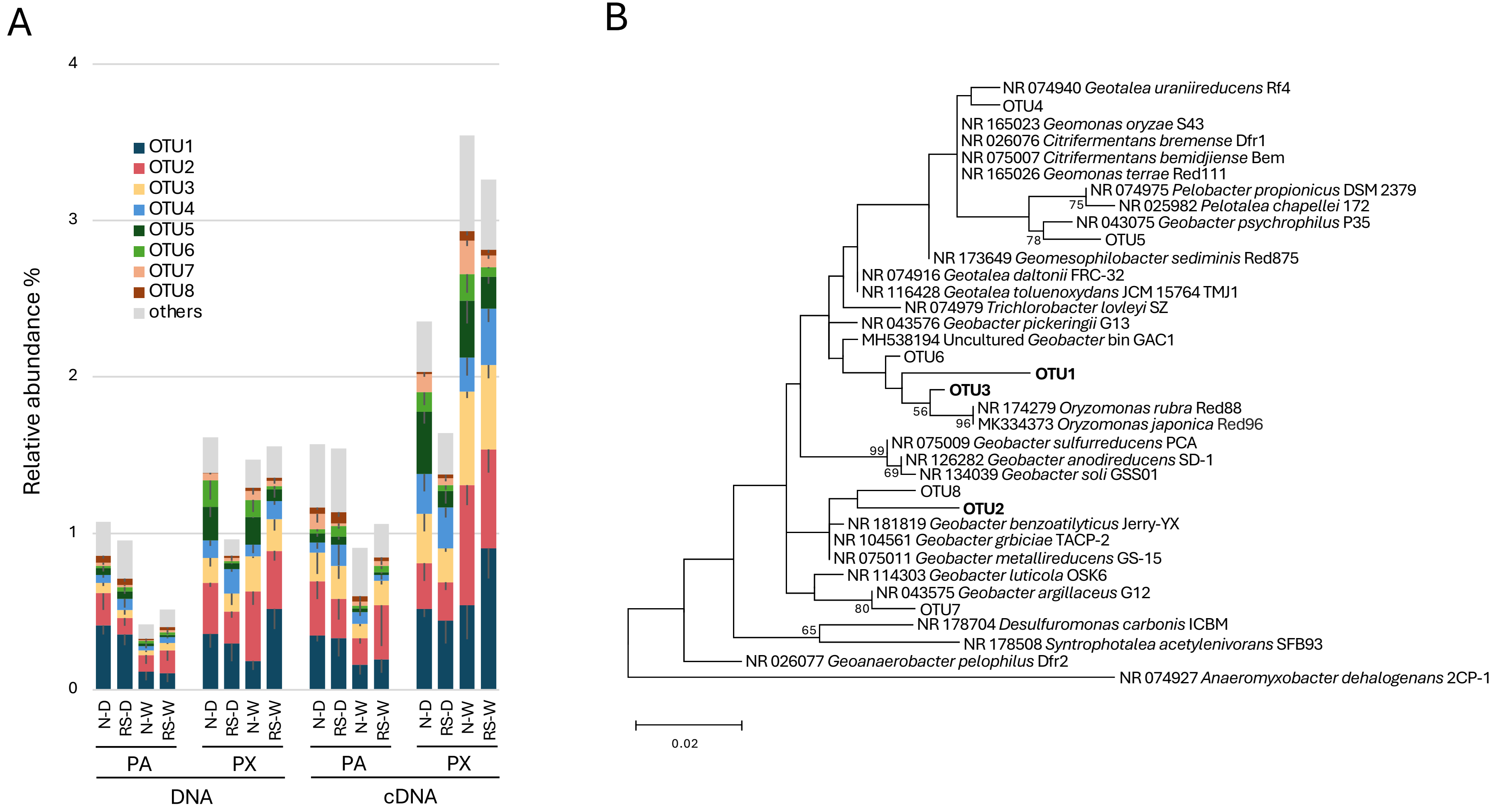

Fig. S4 Relative abundance of the unclassified group of *Geobacteraceae* with OTU ratio (A) and phylogenetic analysis of the partial 16S rDNA sequences of the top 8 most abundant OTUs in the unclassified group of *Geobacteraceae* (B). The relative abundance represents the proportion of total rDNA sequences from each sample. N, rice straw removed; RS, rice straw plowed; D, drained sample; W, water-logged sample. The trees were constructed using the maximum-likelihood method. Bootstrap values (based on resampling 500 times) > 50 are shown.

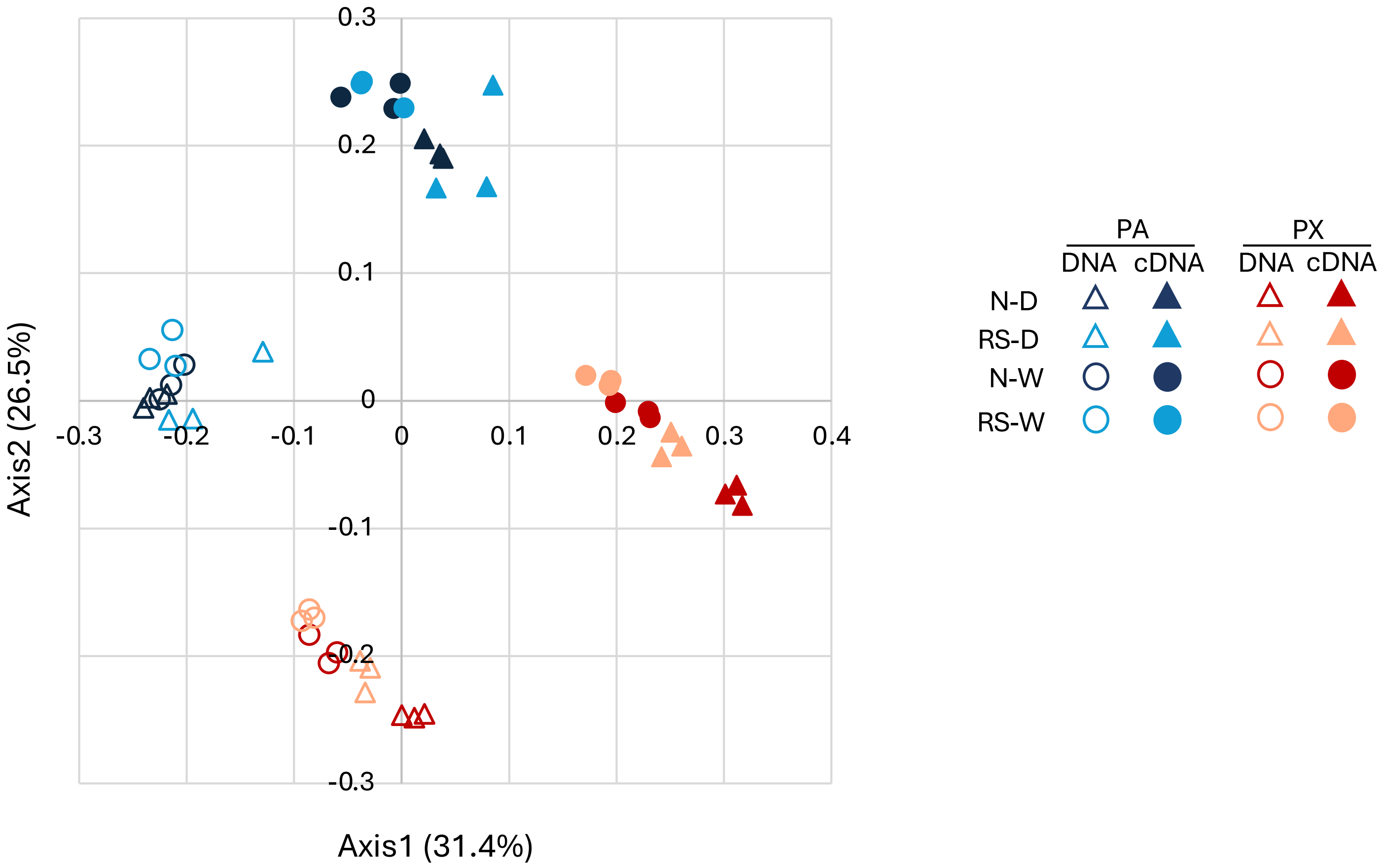

Fig. S5 Principal coordinate analysis (PCoA) plots of 16S rDNA sequences in each sample. N, rice straw removed; RS, rice straw plowed; D, drained sample; W, water-logged sample.

References

Armitage, D., Gallagher, K., and Youngblut, N. (2012) Millimeter-scale patterns of phylogenetic and trait diversity in a salt marsh microbial mat. Front Microbiol 2; 293.

Breidenbach, B., and Conrad, R. (2015) Seasonal dynamics of bacterial and archaeal methanogenic communities in flooded rice fields and effect of drainage. Front Microbiol 5; 752.

Engelbrektson, A., Kunin, V., Wrighton, K., Zvenigorodsky, N., Chen, F., Ochman, H., and Hugenholtz, P. (2010) Experimental factors affecting PCR-based estimates of microbial species richness and evenness. ISME J 4; 642–647.

Holmgren, G. G. S. (1967) A rapid citrate-dithionite extractable iron procedure. Soil Sci Soc Am J, Soil Science Society 31; 210–211.

Ikeda, S., Tsurumaru, H., Wakai, S., Noritake, C., Fujishiro, K., Akasaka, M., and Ando, K. (2008) Evaluation of the effects of different additives in improving the DNA extraction yield and quality from Andosol. Microbes Environ 23; 159–166.

Mori, K., Iino, T., Suzuki, K.-I., Yamaguchi, K., and Kamagata, Y. (2012) Aceticlastic and NaCl-requiring methanogen "*Methanosaeta pelagica*" sp. nov., isolated from marine tidal flat sediment. Appl Environ Microbiol 78; 3416–3423.

Nunoura, T., Oida, H., and Miyazaki, J. (2008) Quantification of *mcrA* by fluorescent PCR in methanogenic and methanotrophic microbial communities. FEMS Microbiol 64; 240–247.

Schloss, P. D., Westcott, S. L., Ryabin, T., Hall, J. R., Hartmann, M., Hollister, E. B., et al. (2009) Introducing mothur: open-source, platform-independent, community-supported software for describing and comparing microbial communities. Appl Environ Microbiol, Am Soc Microbiol 75; 7537–7541.

Shakya, M., Quince, C., and Campbell, J. (2013) Comparative metagenomic and rRNA microbial diversity characterization using archaeal and bacterial synthetic communities. Environ Microbiol 15; 1882–1899.

Tamura, K., Stecher, G., and Kumar, S. (2021) MEGA11: Molecular evolutionary genetics analysis version 11. Mol Biol Evol 38; 3022–3027.

Töwe, S., Wallisch, S., Bannert, A., Fischer, D., and Hai, B. (2011) Improved protocol for the simultaneous extraction and column-based separation of DNA and RNA from different soils. J Microbiol Methods 84; 406–412.

Vaksmaa, A., Jetten, M. S. M., Ettwig, K. F. and Lüke, C. (2017) *McrA* primers for the detection and quantification of the anaerobic archaeal methanotroph "*Candidatus* Methanoperedens nitroreducens". Appl Microbiol Biotechnol 101; 1631–1641.
